# Switch therapy in hospitalized patients with community-acquired pneumonia: Tigecycline vs. Levofloxacin

**DOI:** 10.1186/1471-2334-12-159

**Published:** 2012-07-19

**Authors:** Julio A Ramirez, Angel C Cooper, Timothy Wiemken, David Gardiner, Timothy Babinchak

**Affiliations:** 1 University of Louisville, Louisville, KY, USA; 2 Pfizer Inc, Collegeville, PA, USA; 3 Division of Infectious Diseases, University of Louisville, 501 E Broadway Suite 308, Louisville, KY, 40202, USA

**Keywords:** Tigecycline, Levofloxacin, CAP, Oral switch

## Abstract

**Background:**

Switch therapy is a management approach combining early discontinuation of intravenous (IV) antibiotics, switch to oral antibiotics, and early hospital discharge. This analysis compares switch therapy using tigecycline versus levofloxacin in hospitalized patients with community-acquired pneumonia (CAP).

**Methods:**

A prospective, randomized, double-blind, Phase 3 clinical trial; patients were randomized to IV tigecycline (100 mg, then 50 mg q12h) or IV levofloxacin (500 mg q24h). Objective criteria were used to define time to switch therapy; patients were switched to oral levofloxacin after ≥6 IV doses if criteria met. Switch therapy outcomes were assessed within the clinically evaluable (CE) population.

**Results:**

In the CE population, 138 patients were treated with IV tigecycline and 156 were treated with IV levofloxacin. The proportion of the population that met switch therapy criteria was 67.4% (93/138) for tigecycline and 66.7% (104/156) for levofloxacin. The proportion that actually switched to oral therapy was 89.9% (124/138) for tigecycline and 87.8% (137/156) for levofloxacin. Median time to actual switch therapy was 5.0 days each for tigecycline and levofloxacin. Clinical cure rates for patients who switched were 96.8% for tigecycline and 95.6% for levofloxacin. Corresponding cure rates for those that met switch criteria were 95.7% for tigecycline and 92.3% for levofloxacin.

**Conclusions:**

Switch therapy outcomes in hospitalized patients with CAP receiving initial IV therapy with tigecycline are comparable to those of patients receiving initial IV therapy with levofloxacin. These data support the use of IV tigecycline in hospitalized patients with CAP when the switch therapy approach is considered.

**ClinicalTrials.gov Identifier:**

NCT00081575

## Background

Community-acquired pneumonia (CAP), one of the most common infectious diseases managed by clinicians, is a key source of morbidity, mortality and expenditure of health-care resources [1,2]. Empiric antimicrobial recommendations, aimed at use of “correct-spectrum” coverage, are outlined in guidelines adopted by numerous global agencies [3-10], including evidence-based guidelines from the Infectious Diseases Society of America and the American Thoracic Society (IDSA/ATS) [11]. Yet, a recent compliance evaluation conducted in 36 hospitals in 14 countries found that practitioners do not routinely follow the guidelines; improvement is needed, especially in the areas of CAP prevention, initial empirical therapy, and switch from intravenous (IV) to oral antibiotics [12].

Identifying patients who may be candidates for early switch from IV to oral antibiotic therapy and potentially early hospital discharge is important in the management of CAP [12-14]. Switch therapy is generally considered appropriate and safe when four criteria indicative of clinical stability are met, including improvement in cough and respiratory distress-related symptoms, defervescence of fever for at least 8 hours, normalizing white blood cell count, and adequate oral intake and gastrointestinal tract absorption. The majority of patients with non-severe disease can switch from IV to oral therapy within 2–4 days. Previous prospective studies of hospitalized CAP patients have shown that clinical cure rates with switch therapy were high [15-17], even in patients whose pneumonia is complicated by bacteremia [18]. Switch therapy also has health economic benefits via reduction in costs due to drug administration and decreased length of hospital stay [13].

Tigecycline is a first-in-class expanded broad-spectrum, glycylcycline antibiotic [19], and is approved by the U.S. FDA for the treatment of community-acquired bacterial pneumonia caused by penicillin-susceptible *Streptococcus pneumoniae*, beta-lactamase negative *Haemophilus influenzae*, and *Legionella pneumophila*. Tigecycline is well distributed into lung tissues and fluids [20], and has been shown to be as effective as levofloxacin in two global Phase 3 clinical trials of CAP [21].

In the current analysis, data from one of the pivotal prospective trials that compared the efficacy of tigecycline vs. levofloxacin in hospitalized patients with CAP [22] was used to assess outcomes in patients switched to oral therapy. The primary objectives of this study were 1) to compare clinical cure rates for hospitalized patients with CAP treated empirically with IV tigecycline followed by a switch to oral levofloxacin versus those treated empirically with IV levofloxacin followed by a switch to oral levofloxacin and 2) to compare time to switch therapy for hospitalized patients with CAP treated empirically with IV tigecycline versus those treated empirically with IV levofloxacin.

## Methods

### Study design and treatment

A detailed description of the patients and methodology for tigecycline study 308 are described elsewhere [22]. In brief, a Phase 3, multicenter, randomized, double-blind (third party unblinded) clinical trial was conducted to compare the efficacy and safety of tigecycline with levofloxacin in hospitalized adults with CAP. The current analysis focused on comparing efficacy outcomes for two subgroups of patients: those receiving oral switch therapy and those not switched to an oral antibiotic.

Patients were randomly assigned (1:1) to initially receive either IV tigecycline (100 mg initially followed by 50 mg every 12 h) or IV levofloxacin (500 mg every 24 h) [22]. A reduced dose of levofloxacin was given to patients with impaired renal function. After at least 3 days of IV therapy (6 IV doses), patients in either treatment group could be switched to oral levofloxacin treatment (500 mg every 24 h), at the investigator's discretion, after meeting specified criteria (see below). The total duration of study therapy was 7 to 14 days at the investigator’s discretion.

### Patient eligibility

Hospitalized adult patients (at least 18 years of age) of either gender with CAP who initially required IV antibiotics could participate. Each patient had a documented fever within 24 h of enrolment, a new lung infiltrate confirmed by chest radiograph within 48 h of receiving the first dose of study medication and at least 2 common signs and/or symptoms suggestive of CAP (e.g., cough, production of purulent sputum, WBC >10 × 10^9^/L). Patients were not allowed to participate in the study for any of the following reasons: recent hospitalization within 14 days, residence in a long-term care facility for ≥14 days before onset of symptoms, required treatment in the ICU at the time of randomization, and those with known or suspected *Pseudomonas aeruginosa* infection.

### Oral switch criteria

In study 308, the specific criteria listed below were to be satisfied at the time the patient was switched to oral levofloxacin. The improvements in signs and symptoms of pneumonia (e.g., cough, shortness of breath, temperature) were to be interpreted by the investigator in relation to the prior day, not baseline. The specific criteria, as judged by the investigator, indicating that clinical stability had been achieved and that the patient was a candidate for oral therapy included the following:

1. Cough and shortness of breath improving.

2. Patient afebrile for ≥24 hours (oral temperature <37.8°C/100°F, or equivalent for axillary, tympanic, or rectal/core temperature), on 2 or more measurements at least 24 hours apart, with no known spiking of temperature during that interval.

3. White blood cell count improving (no change was required if the WBC count was within normal range): >10% decrease if initial WBC count was elevated (>10 × 10^9^/L); >5% decrease in immature neutrophils (bands) if initially >15%; or >10% increase if patient was initially leukopenic (WBC <4.5 × 10^9^/L).

4. Oral intake and gastrointestinal tract absorption adequate.

Oral intake was defined as a patient taking food by mouth without gastrointestinal intolerance on any day. For the other three objective criteria of WBC count, cough/dyspnea, and temperature, the patient was considered to reach criteria based on change from baseline or the prior day. In this analysis, patients had to meet all three objective criteria and oral intake criteria to be considered a candidate for oral therapy. For WBC, patients could meet any of the criteria below, up to the last day of therapy visit:

· Previous WBC is normal and first WBC value post-baseline is normal

· Previous WBC is above normal and WBC decreases by 10% (second value)

· Previous WBC is below normal and WBC increases by 10% (second value)

· If previous bands >15% and bands decrease by 5% (second value)

For cough and dyspnea, patients could meet any of the criteria below from the first visit up to last day of therapy visit:

· Cough and dyspnea are absent

· Cough and dyspnea improve from the previous visit (second visit)

· Cough and dyspnea are unchanged from previous visit and improved from baseline

For temperature, patient met criteria if temperature was <37.8 °C with a previous temperature <37.8 °C (second visit). If multiple temperatures were taken during same relative day, the maximum temperature was used.

### Time to clinical stability

Patients were considered to have clinical improvement on the day they met all 4 criteria for switch therapy according to the study protocol. Patients who satisfied the criteria for switch therapy during the first 3 days of hospital treatment were considered to have early clinical stability. Patients who met the criteria for switch therapy from days 4 to 7 of hospital treatment were considered to have late clinical stability.

### Analysis population and efficacy assessments

The present analysis is restricted to patients categorized as clinically evaluable (CE). Patients who had clinical evidence of CAP by meeting the minimal disease criteria were considered to be CE if they satisfied inclusion and exclusion criteria, received no more than one dose of a non-once, daily, non-study antibacterial agent (single agent or combination therapy) to treat the current episode of CAP before the first dose of study drug, did not receive other concomitant systemic antimicrobial therapy unless a treatment failure, received at least 2 full days of study drug if clinical failure or 5 full days of study drug (IV plus oral) if clinical cure, were adherent with therapy (i.e., ≥80%, but ≤120% of medication administered), had an assessment of cure or failure (and not indeterminate) at the test-of-cure (TOC) visit, and the study blind was maintained. The primary efficacy endpoint was clinical response in the CE population at the TOC visit.

### Statistical analysis

Categorical baseline demographic and medical variables were analyzed using Fisher’s exact test and chi-square test. Continuous variables were compared using a one-way analysis of variance (ANOVA) model with treatment as a factor. The time to meet oral switch criteria was analyzed by the Kaplan-Meier approach using log-rank test for differences in survival curves.

Overall clinical response rates were compared for hospitalized patients with CAP treated empirically with IV tigecycline followed by a switch to oral levofloxacin versus those treated empirically with IV levofloxacin followed by a switch to oral levofloxacin, using Fisher’s exact test. Clinical response was also evaluated for each treatment for patients in the following groups: *IV/PO Switch* (all patients who switched to PO therapy) versus *IV-only* (patients not switched to PO therapy) and *Met Switch Criteria* (regardless of whether the patient was switched to oral therapy) versus *Did Not Meet Switch Criteria*. *P* values <0.05 were considered significant. Statistical analysis was performed by the Global Biostatistics and Programming department of Wyeth Research, Collegeville, PA, which was acquired by Pfizer Inc in October of 2009.

The study protocol was reviewed and approved by each investigator's independent ethics committee or institutional review board in accordance with local regulations and good clinical practices. Written informed consent was obtained from each patient or his or her guardian before initiation of any study procedure according to the guidelines of each institution.

## Results

### Patients and treatment

A total of 425 patients were available for the Intent To Treat population analysis in study 308. A total of 294 patients were included in the CE population. The CE population was comprised of 138 hospitalized patients with CAP treated empirically with IV tigecycline and 156 hospitalized patients with CAP treated empirically with IV levofloxacin (Table 1). Nearly two-thirds (67.0%; [197/294]) of patients met objective IV to oral switch criteria per study protocol (93/138, 67.4% tigecycline and 104/156, 66.7% levofloxacin; Table 1). Switch to oral therapy was performed in 88.8% (261/294) of patients (124/138, 89.9% tigecycline; 137/156, 87.8% levofloxacin; Table 1). Demographic and baseline medical characteristics, grouped by patients who were switched or not switched to oral therapy, are outlined in Table 2.

**Table 1:** Clinically Evaluable Population

**Number of patients, n (%)**	**Tigecycline (N = 138)**	**Levofloxacin (N = 156)**	**Total (N = 294)**
Patients switched to oral therapy	124 (89.9%)	137 (87.8%)	261 (88.8%)
Patients not switched to oral therapy	14 (10.1%)	19 (12.2%)	33 (11.2%)
Patients meeting switch criteria as determined by present analysis	93 (67.4%)	104 (66.7%)	197 (67.0%)
Patients not meeting switch criteria as determined by present analysis	45 (32.6%)	52 (33.3%)	97 (33.0%)
Patients switched without meeting switch criteria as determined by present analysis	31 (22.5%)	33 (21.2%)	64 (21.8%)

**Table 2:** Baseline Demographic and Medical Characteristics for CE Population: Switched vs. Not Switched from IV to Oral Therapy

**Characteristic**	**Switched (IV to oral)**			**No Switch (IV only)**		
	**Tigecycline N = 124**	**Levofloxacin N = 137**	***P*-value**	**Tigecycline N = 14**	**Levofloxacin N = 19**	***P*-value**
**Age, yrs, mean ± SD**	55.2 ±16.4	54.0 ± 20.4	0.613	60.1 ± 18.1	63.1 ± 17.2	0.634
**Male, n (%)**	64 (51.6)	83 (60.6)	0.145	9 (64.3)	13 (68.4)	0.803
**Ethnic origin, n (%)**			0.189			0.620
White	80 (64.5)	87 (63.5)		9 (64.3)	9 (47.4)	
Hispanic	29 (23.4)	34 (24.8)		5 (35.7)	7 (36.8)	
Black	14 (11.3)	9 (6.6)		0	1 (5.3)	
Asian	0	4 (2.9)		0	1 (5.3)	
Other	1 (0.8)	3 (2.2)		0	1 (5.3)	
**Fine Score, n (%)**			0.452			0.816
I	28 (22.6)	41 (29.9)		3 (21.4)	2 (10.5)	
II	43 (34.7)	42 (30.7)		1 (7.1)	1 (5.3)	
III	33 (26.6)	29 (21.2)		5 (35.7)	7 (36.8)	
IV	20 (16.1)	25 (18.2)		5 (35.7)	9 (47.4)	
**Prior antibiotic failure, n (%)**	2 (1.6)	4 (3.0)	0.471	0	1 (5.3)	0.383
**Therapy duration, days, mean ± SD**	11.39 ± 2.0	11.26 ± 2.0	0.599	6.7 ± 3.1	6.3 ± 3.7	0.746
**Presence of multilobar infiltrates, n (%)**	32 (25.8)	22 (16.1)	-	7 (50.0)	7 (36.8)	-
**Presence of altered mental status, n (%)**	2 (1.6)	5 (3.6)	-	0	1 (5.3)	-

**Figure 1:**
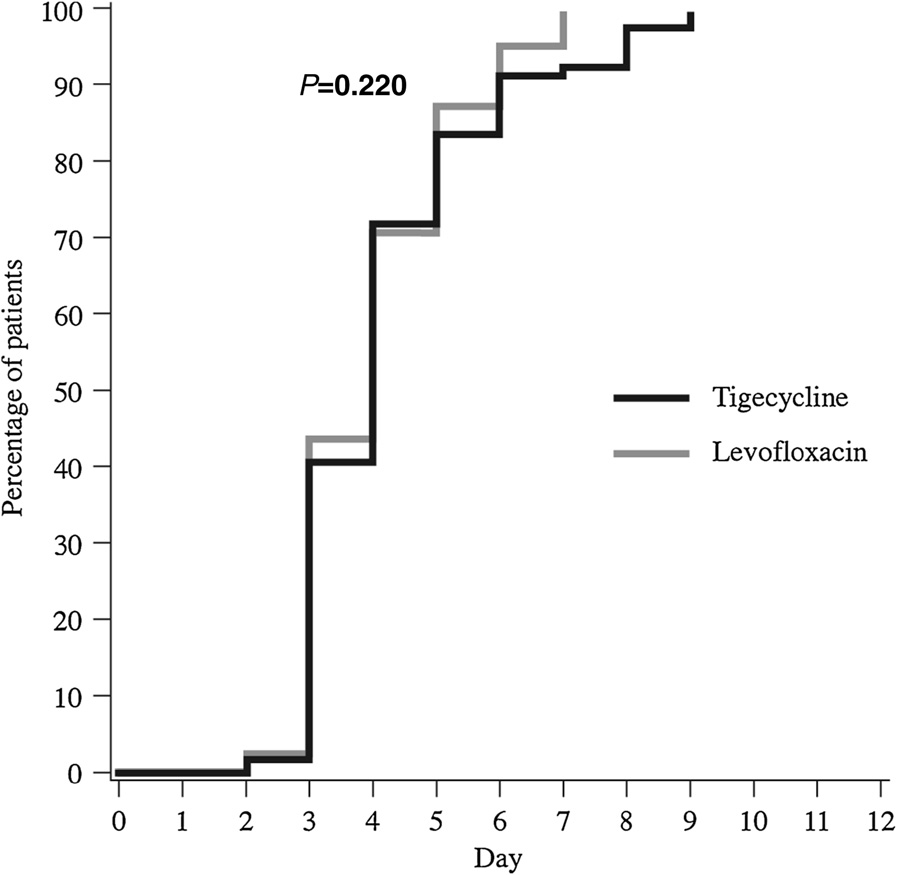
Days until patients met objective criteria for IV to oral switch.

**Figure 2:**
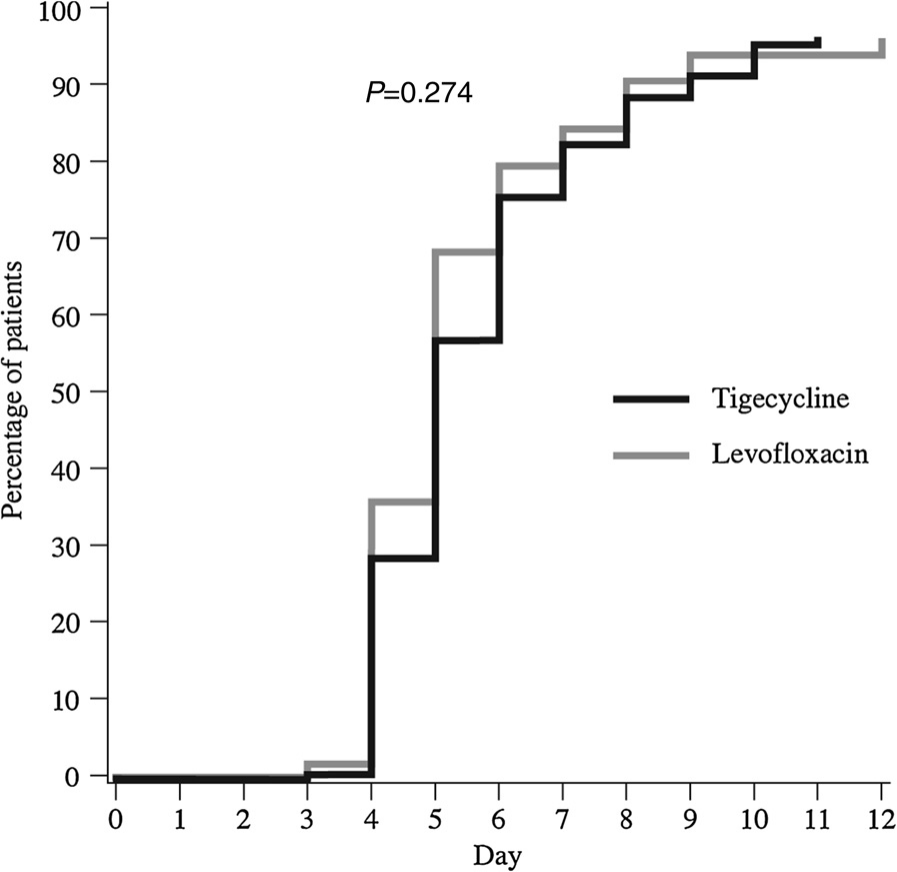
Days until patients actually switched from IV to oral therapy.

**Figure 3:**
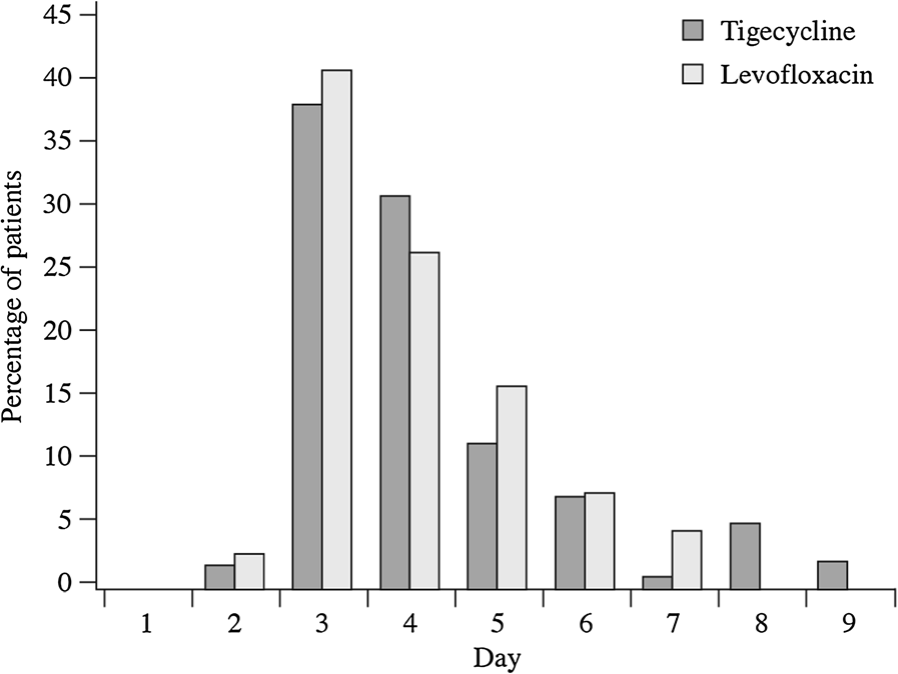
Number of patients meeting objective IV to oral switch criteria.

The median time to clinical stability (i.e., time when all criteria for oral switch were met) for the CE population was 4 days each for tigecycline and levofloxacin (*P* = 0.220; Figure 1). Nearly 50% of patients in both treatment groups were categorized as having early clinical stability. The median time to actual switch to oral therapy was 5.0 days each for tigecycline- and levofloxacin-treated patients (*P* = 0.274; Figure 2). Figure 3 illustrates the number of patients in each group who met IV to oral switch criteria, as determined by the present analysis, by treatment day. Of note, 64 patients who were switched to oral therapy (31 tigecycline, 33 levofloxacin) had not met objective criteria for IV to oral switch, as determined by the present analysis. Thirteen patients who met objective criteria for IV to oral switch were not switched to oral therapy (4 tigecycline, 9 levofloxacin). Although the study protocol had easy to follow and well-defined switch therapy criteria, in 77 of the 294 patients evaluated (26%), the switch therapy criteria were not followed by the investigators.

### Clinical efficacy

**Figure 4:**
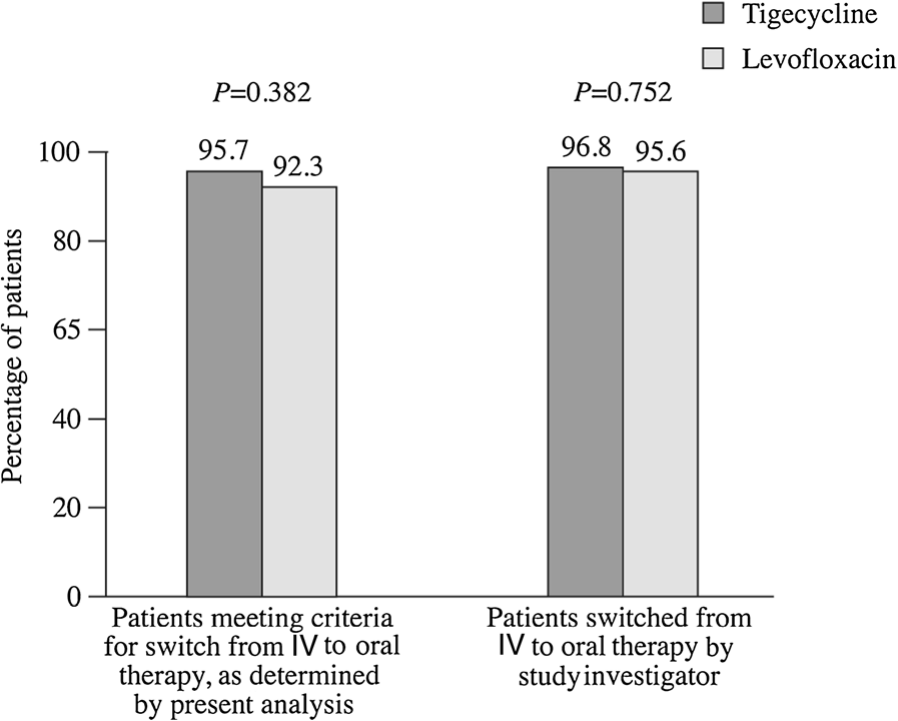
Clinical cures.

For patients switched to oral therapy by the investigator, overall cure rates in the CE population were similar between tigecycline and levofloxacin (tigecycline 96.8% [120/124]; levofloxacin 95.6% [131/137]) (*P* = 0.752; Figure 4). In patients not switched, cure rates were low, as expected, in both treatment groups (tigecycline 35.7% [5/14]; levofloxacin 26.3% [5/19]) (*P* = 0.707). Among patients who met switch criteria as determined by the study protocol, the clinical cure rate was 95.7% (89/93) for tigecycline and 92.3% (96/104) for levofloxacin (*P* = 0.382; Figure 4). Corresponding rates for patients who did not meet switch criteria were 80.0% (36/45) and 76.9% (40/52), respectively (*P* = 0.807).

## Discussion

This study indicates no significant difference in the clinical cure rates for hospitalized patients with CAP treated empirically with IV tigecycline followed by a switch to oral levofloxacin versus those treated empirically with IV levofloxacin followed by a switch to oral levofloxacin. There was also no significant difference in the time to switch therapy for hospitalized patients with CAP treated empirically with IV tigecycline versus those treated empirically with IV levofloxacin. The median time to meet criteria for switch therapy was 4 days for both study groups. Our study findings indicating equal clinical activity and equal time to switch therapy for each study arm suggest that both antibiotics have similar activity against pulmonary pathogens.

Nearly half of all hospitalized patients with CAP met criteria for switch therapy by day 3 of hospitalization. These data indicating early clinical stability in a significant number of hospitalized patients with CAP, are similar to those reported in prior studies on CAP [15-18]. It is important to recognize these groups of patients with early clinical improvement since they can be targeted for an approach for early switch therapy and early hospital discharge. National guidelines indicate that patients can be safely switched to oral therapy on the same day they meet criteria for clinical stability. In our study, the median time for patients to meet criteria for clinical stability was 4 days in both study arms, but the time to actual switch was 5 days in both study arms. This one-day delay to implement oral switch will not offer any clinical benefit and is likely to delay hospital discharge. A delayed switch to oral therapy may also be associated with a number of potential detrimental effects to the patient, the caregiver, and the healthcare system. Although our analysis did not capture these parameters, delayed hospital discharge may increase the patient's risk for nosocomial infections [16].

Our data indicate that patients who were less severely ill (e.g., Fine Score I-III) were more likely to reach criteria for switch therapy. These findings are in agreement with a recent evaluation of severity of disease at time of hospitalization and time to clinical stability for patients with CAP [23]. The authors found that as severity of disease increased, measured by CRB-65 and Fine Score, time to clinical stability also increased.

This study has several limitations. Although the protocol defined objective criteria for switch therapy, the actual switch to oral therapy was at the discretion of the investigator. The fact that, in 26% of the patients the investigators failed to follow the switch therapy criteria as defined in the protocol, has the potential to induce selection bias. As explained in the Methods section, we used only the clinically evaluable population for our analysis. Since we did not use the Intent To Treat population, this may have incorporated some bias into the results. The study did not collect long-term efficacy data beyond the test of cure visit at 7-23 days after the last day of antibiotic therapy. Because of the short follow-up in some patients there is a possibility for missing cases with relapse of infection after switch. In this study, we evaluated the role of two antibiotics in time to clinical stability. Confounding factors for this association include the bacterial etiology of CAP as well as host factors. The generalizability of our data is limited due to the fact that patients admitted to the ICU or patients with risk factors for *Pseudomonas aeruginosa* were excluded from the trial. One strength of this study is that randomization into the two study arms may limit confounding effects.

Because duration of hospitalization is an important driver in the costs of CAP, implementing an oral switch protocol will provide economic benefits. Although not all patients who are candidates for oral switch therapy can be discharged early, adoption of universal switch therapy in the U.S. is estimated to reduce hospital days by more than a half a million days annually with an overall savings in excess of $400 million [13]. Adoption of an oral switch strategy is also an important component of antibiotic stewardship programs, which are aimed, in part, at minimizing emergence of multiple-drug resistant bacteria [24-26]. Despite data that support the efficacy of oral switch therapy [15-18, 27-30], practitioners often fail to assess if a patient is an appropriate candidate, or elect to continue IV therapy because the patient is improving.

## Conclusion

In conclusion, the tigecycline approach to switch therapy is equivalent to a levofloxacin approach to switch therapy. This study supports the use of short-course tigecycline in hospitalized patients with community-acquired pneumonia followed with an early switch to oral therapy in appropriate patients.

## Competing interests

AC is an employee of Pfizer Inc. DG and TB are former employees of Pfizer, Inc. JAR is on the Speakers’ Bureau for Pfizer Inc. TW has no conflicts to report.

## Author contributions

JAR: Contributed to protocol development, data analysis, manuscript development and critical review. ACC: Contributed to protocol development, data analysis, manuscript development and critical review. TW: Contributed to data analysis, manuscript development and critical review. DG: Contributed to protocol development, data analysis, manuscript development and critical review. TB: Contributed to protocol development, data analysis, manuscript development and critical review. All authors read and approved the final manuscript.

## Pre-publication history

The pre-publication history for this paper can be accessed here:

http://www.biomedcentral.com/1471-2334/12/159/prepub
